# Isthmocele: From Risk Factors to Management

**DOI:** 10.1055/s-0038-1676109

**Published:** 2019-01-15

**Authors:** Piergiorgio Iannone, Giulia Nencini, Gloria Bonaccorsi, Ruby Martinello, Giovanni Pontrelli, Marco Scioscia, Luigi Nappi, Pantaleo Greco, Gennaro Scutiero

**Affiliations:** 1 Section of Obstetrics and Gynecology, Department of Morphology, Surgery and Experimental Medicine, Azienda Ospedaliero-Universitaria S. Anna, Università di Ferrara, Cona, Ferrara, Italy; 2 Section of Obstetrics and Gynaecology, Policlinico di Abano Terme, Abano Terme, Padova, Italy; 3 Department of Medical and Surgical Sciences, Institute of Obstetrics and Gynecology, University of Foggia, Foggia, Italy

**Keywords:** isthmocele, niche, cesarean section scar defect, hysteroscopy, laparoscopy, istmocele, nicho, defeito cicatricial de cesariana, histeroscopia, laparoscopia

## Abstract

**Objective** The aim of the present study was to perform a comprehensive review of the literature to provide a complete and clear picture of isthmocele—a hypoechoic area within the myometrium at the site of the uterine scar of a previous cesarean section—by exploring in depth every aspect of this condition.

**Methods** A comprehensive review of the literature was performed to identify the most relevant studies about this topic.

**Results** Every aspect of isthmocele has been studied and described: pathophysiology, clinical symptoms, classification, and diagnosis. Its treatment, both medical and surgical, has also been reported according to the actual literature data.

**Conclusion** Cesarean section is the most common surgical procedure performed worldwide, and one of the consequences of this technique is isthmocele. A single and systematic classification of isthmocele is needed to improve its diagnosis and management. Further studies should be performed to better understand its pathogenesis.

## Abstract (pt)

**Resumo:**

**Objetivo** O objetivo do presente estudo foi realizar uma revisão abrangente da literatura a fim de fornecer um quadro completo e claro da istmocele—uma área hipoecoica dentro do miométrio no local da cicatriz uterina de uma cesariana anterior—aprofundando todos os aspectos desta condição

**Métodos** Uma revisão abrangente da literatura foi realizada para identificar os estudos mais relevantes sobre este tema.

**Resultados** Todos os aspectos da istmocele foram estudados e descritos: fisiopatologia, sintomas clínicos, classificação e diagnóstico. Os tratamentos médico e cirúrgico também foram relatados de acordo com os dados reais da literatura.

**Conclusão** A cesárea é o procedimento cirúrgico mais comum realizado em todo o mundo, e uma das consequências desta técnica é a istmocele. Uma classificação única e sistemática da istmocele é necessária para melhorar seu diagnóstico e manejo. Novos estudos devem ser realizados para melhor entender sua patogênese.

## Introduction

Cesarean section (CS) is one of the most common surgical operations performed worldwide.^1^ Nevertheless, the percentage of CS deliveries has dramatically increased in most developed countries in the last decades, which has given rise to a great concern.^2^ ^3^ According to the latest data from 150 countries, CS rates range from 6 to 27.2%.^3^ ^4^ A higher maternal socioeconomic status seems to be associated with a greater likelihood of CS.^5^ The World Health Organization (WHO) states that the optimal CS rate is around 15%.^6^ Cesarean incisions usually heal without consequences, but there is always the possibility of complications. Lately, the increasing rate of CSs has increased the interest in the short- and long-term morbidity of cesarean scar defect.^7^

Cesarean scar defect—also called isthmocele, niche, diverticulum or pouch—was first described by Poidevin in 1961^8^ as a wedge-shaped defect in the uterine wall. Due to the variety of names, we prefer to refer to this defect as isthmocele, which, we think, gives a better idea of the anatomical defect described.

Isthmocele can be defined as a hypoechoic area within the myometrium of the lower uterine segment, reflecting a discontinuation of the myometrium at the site of the uterine scar of a previous CS.^6^ ^7^

Bij de Vaate et al^9^ defined isthmocele as an anechoic area at the site of the cesarean scar with a depth of at least 1 mm. The prevalence of isthmocele is difficult to quantify, ranging between 24 and 70% using transvaginal ultrasound, and between 56 and 84% using sonohysterography (SHG).^1^ ^10^ In > 50% of the women with a history of CS, isthmocele can be observed when examined by SHG between 6 and 12 months after the CS.^7^ Cesarean section defects can be asymptomatic. However, in many cases, they can lead to a series of gynecological symptoms, such as abnormal uterine bleeding, dysmenorrhea, chronic pelvic pain, dyspareunia, and infertility_***.***_ 1 6 11 They may also be responsible for future obstetrical complications, such as ectopic pregnancy, uterine rupture, and placental anomalies (for example, placenta accreta).^11^ The objective of the present review was to give a wide and complete overview of the current literature by describing every aspect of this condition, deeply analyzing its risk factors, its diagnosis, and its surgical and medical management.

## Methods

A review of the literature was conducted to identify the most relevant studies reported in the English language. We have searched the PubMed MEDLINE electronic database, the International Prospective Register of Systematic Reviews (PROSPERO) database, the Cochrane Database, the Centre for Reviews and Dissemination (CRD) database, the Database of Abstracts of Reviews of Effects (DARE), and the National Institute for Health Research (NHS) database and studied all the articles published until October 2017. The keywords used were: *isthmocele*, *niche*, *cesarean section defect*, *cesarean section scar*, *cesarean section diverticulum*, and *cesarean section pouch*. Different combinations of the terms were used. The filters used were studies conducted in humans, systematic reviews, trials, meta-analyses, and multicentric trials. Moreover, the references in each article were searched in order to identify potentially missed studies.

## Results

**Fig. 1:**
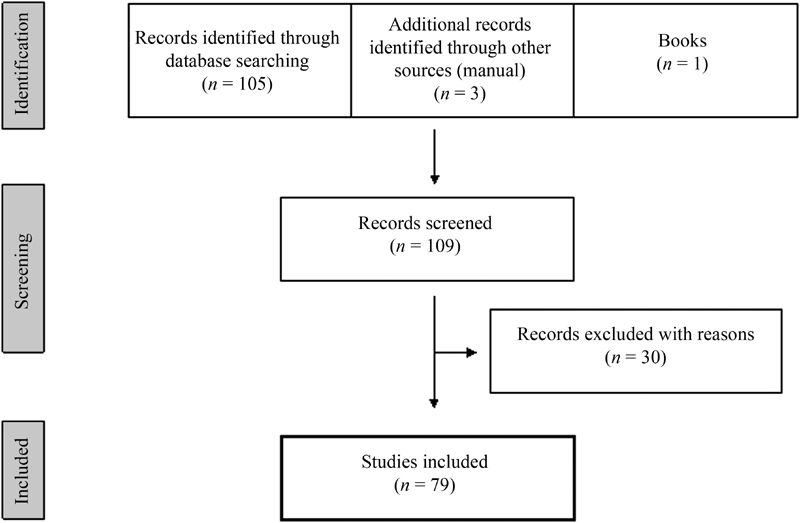
Flowchart of the article selection process.

The research led to the retrieval of 105 articles; other 3 articles and 1 book were added manually. Thirty articles were excluded from our research. The exclusion criteria were: articles not in English, not relevant to the review, and abstracts. Fig. 1 shows the selection process.

**Fig. 2:**
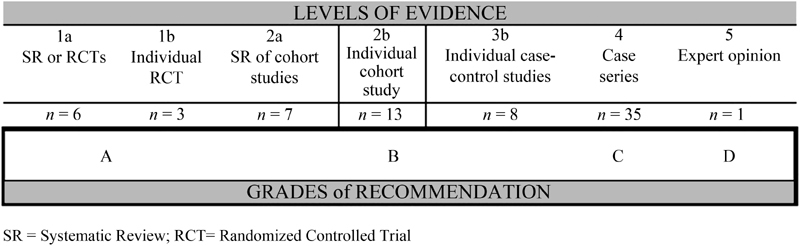
Classification of studies according to Oxford Centre for Evidence et al. based Medicine.

We have divided the articles following the grades of recommendations and levels of evidence proposed by the Oxford Centre for Evidence-Based Medicine, as shown in Fig. 2.

## Discussion

### Pathophysiology

The pathophysiology of the development of isthmocele is still unclear, although many authors have studied the associated risk factors.^2^ ^6^ ^11^ However, data available in the current literature are very poor due to the lack of evidence. Nevertheless, the pathophysiology may be related both to the surgery technique and to patient factors.

### Risk Factors: Surgery and Patient

#### Surgery Technique Factors

Very low uterine incisions are reported to be independent risk factors for the development of isthmocele.^12^ A higher prevalence of CS defects has been observed in those patients with a CS performed during active labor with cervical effacement.^13^ Vikhareva Osser et al^14^ described an increased development of isthmocele in case of a cervical dilatation > 5 cm or of a labor duration of > 5 hours. Moreover, isthmocele was observed in the upper two-thirds of the cervix in women with an elective CS, while, in the case of CSs performed after cervical dilatation, the niche was in the lower part of the cervical canal.^15^ An explanation to this phenomenon might be that lower incisions through the cervical tissue, which contains mucus-producing glands, might interfere negatively with the wound healing process.^6^

Another plausible factor is the closure technique, that is, double- versus single-layer closure.^2^ ^6^ ^7^ These techniques vary among countries and have changed over the years. For example, in some European countries, such as Belgium and the Netherlands, the single-layer closure technique is the most performed, while in the United Kingdom, double-layer closure is the recommended technique.^6^ ^16^ The CORONIS^17^ and the CAESAR^18^ trials evaluated maternal outcomes after 6 weeks in patients undergoing CS with either the double- or the single-layer technique. Both trials observed no significant differences in terms of maternal outcomes in both surgical methods. A 2014 review by Roberge et al^19^ also found no difference in the development of scar defects among the techniques used. A recent research also observed that the incidence of cesarean scar formation and niche depth was independent of the hysterotomy closure technique used.^20^ In a recent meta-analysis, Di Spiezio Sardo et al^7^ reported that women who received a single-layer uterine closure had a similar incidence of uterine scar defects as women who received a double-layer closure.

Ceci et al,^21^ however, observed that patients who received a locked continuous single-layer suture compared with the interrupted single-layer suture group showed a defect area statistically larger on ultrasound and hysteroscopy evaluation, probably due to an ischemic effect on the uterine tissue.^21^ ^22^

The hypothesis might be that not closing the deeper muscular layer leads to a disrupted myometrium and to the development of isthmocele.^6^ However, due to missing data, a specific surgical technique for uterine closure cannot be recommended yet.^7^

Another proposed hypothesis is the surgery itself.^6^ It is well known that surgery can lead to the development of adhesion, and that many factors may influence this process, such as inflammation, tissue ischemia, tissue manipulation, and inadequate hemostasis.^23^ The formation of adhesion between the CS scar and the abdominal wall might be a cause of the development of isthmocele. Vervoort et al^6^ hypothesized that the retraction of the scar tissue might pull the uterine scar toward the abdominal wall, inducing the development of isthmocele.

#### Patient Factors

Patient factors may play a role in isthmocele and in the CS healing process, due to individual differences.^6^ Some studies have observed the association between the development of scar defects and patient factors, such as retroflexed uterus, multiple CSs, body mass index (BMI), and hypertension, but its mechanism of action remains unclear.^2^ ^6^

We still do not know why some patients develop caesarean scar defects while others do not. Probably, a single individual genetic predisposition along with other unknown factors might be the key to this phenomenon. Further studies are needed to answer this question.

### Clinical Symptoms

As first described by Morris in 1995,^24^ cesarean scar defects may be associated with clinical symptoms. The most frequent complaints reported by the patients is abnormal uterine bleeding (AUB), in particular postmenstrual spotting.^11^ ^25^ Abnormal uterine bleeding was found to be present in between 28.9 and 82% of the studied cases, and it seems that there is a correlation between the size of the cesarean defect and the symptoms.^1^ ^25^ ^26^ The pathogenesis of AUB following the development of isthmocele remains unexplained.^27^ It has been hypothesized that menstrual blood can accumulate in the pouch defect and then seep out slowly over the days after menses.^11^ Thurmond et al^28^ also suggested that the disorder results from impaired uterine contractility at the scar area. Several authors have reported and observed a connection between isthmocele, dysmenorrhea, and pelvic pain. The presence of these symptoms might be related to the size of the defect.^1^ Some plausible explanations have been proposed, as the presence of lymphocytic infiltration and anatomical distortion, or of abnormal myocontracture due to the continuous efforts of the uterus to empty the contents of the isthmocele.^11^ ^25^

Infertility may also represent a big issue for patients with CS defects. The lower fertility rate might be related to the persistence of menstrual blood in the pouch, which affects the cervical mucus, as well as sperm motility and implantation.^2^ ^25^ Infertility might also be caused by an inflammatory condition, as it is already known in pathologies characterized by chronic inflammatory states and oxidative stress, such as endometriosis or endometritis.^29^ ^30^ ^31^ In isthmocele, the presence of residual menstrual blood might also lead to an environment of chronic inflammation, thus affecting fertility.^11^

Even though it is rare, isthmocele might lead to the formation of an abscess due to the collection of mucus and menstrual blood, which act as an infection-promoting factor.^32^ Another reported complication is caesarean scar ectopic pregnancy, with an incidence of ∼ 1 in 1,886 to 2,216 pregnancies.^25^ With the development of the fetus and of the gestational sac, the walls of the isthmocele might rupture, leading to the known severe complications related to an ectopic pregnancy.^11^ ^25^

### Classification and Diagnosis

Since 1990, when first described by Chen et al,^33^ ultrasound, in particular transvaginal ultrasound (TVUS) has been used to evaluate caesarean scar defects. Nowadays, TVUS can be considered the most common initial technique to identify isthmocele in patients with a history of previous CS.^11^ Some authors suggest that the ideal moment to perform this diagnostic exam is during the early follicular phase, assuming that the endometrium may improve the identification of the defect and the measurement of its depth and size.^9^

Isthmocele has been reported with several descriptions: as a triangular anechoic area, as a filling defect on the anterior isthmus, or as cystic mass between the lower uterine segment and the bladder.^11^ ^34^ Bij de Vaate et al^9^ proposed a more systematic classification using six shapes to describe the defect: triangle, semicircle, rectangle, circle, droplet, and inclusion cysts. In this study, during a TVUS exam, the uterus was examined for isthmocele: position, length, width, depth, and residual myometrium were recorded. A depth of at least 1 mm is the vertical distance between the base and apex of the defect.^9^ The residual myometrium is the vertical distance between the serosal surface of the uterus and the apex of the defect.^9^ ^11^

The residual myometrium thickness is the most useful discriminating measurement in the evaluation of isthmocele.^35^ The residual myometrium appears thinner on ultrasound in women who received a single-layer closure compared with those who received a double-layer closure.^7^ Moreover, the scar is less thick in patients with two or more previous CSs, and thicker in patients who underwent the last of the previous CS > 2 years earlier.^36^ Large scar defects have been linked to an increased risk of uterine rupture, although the real risk of isthmocele remains unexplained.^37^ Some authors have proposed a cutoff of residual myometrium for risk of uterine rupture, varying between 2.5 mm and 3 mm.^35^

Another important concept is the deficiency degree, introduced by Ofili-Yebovi et al,^38^ which is described as the ratio between the myometrial thickness at the scar and the thickness of the adjacent myometrium. A deficiency rate of < 50% was described as severe.^38^

Since first described by Zilberman et al,^39^ saline infusion SHG has been widely used to assess the uterine cavity in patients with suspected endometrial or intracavitary disease for whom the TVUS might not give a defined diagnosis.^39^ ^40^ Moreover, SHG increases the sensitivity and the specificity for the detection of CS scars by enhancing the defect.^11^ The prevalence of cesarean scar defects in randomly selected women appears to be higher in SHG compared with in TVUS (56–84% versus 24–70%), and the defect seems to be deeper or larger in the SHG.^1^ ^25^ The increased prevalence and scar size, when using SHG, is due to an exaggeration of the size of the defect caused by the increased intrauterine pressure.^1^

Isthmocele may also be diagnosed by hysterosalpingography as an extension of contrast into the myometrial defect at the site of a previous cesarean hysterotomy.^11^ Magnetic resonance imaging (MRI) represents another valuable tool that may be helpful to diagnose and characterize isthmocele.^11^ ^25^ Magnetic resonance imaging is useful to evaluate the thickness of the lower uterine segment, the depth of the isthmocele, and the content of the endometrial and defect cavities.^25^

This imaging method, MRI, may clearly define the defect and enable a faster diagnosis when patients complain of otherwise unexplainable AUB.^11^ ^41^

### Treatment

The treatment of isthmocele is performed to relieve symptoms. Consequently, the asymptomatic cases should not be treated.^42^ The treatment can be medical, although surgery is the most common treatment of choice, based on different approaches: hysteroscopy, laparoscopy (including robotic laparoscopy), laparotomy, vaginal repair, and combined techniques. As it is well known, every surgical treatment has its own specific complications, such as infections, bladder and bowel injuries, and hemorrhage.^43^

#### Medical Treatment

Many authors describe the effectiveness of oral contraceptives in reducing bleeding disorders correlated to isthmocele.^44^ ^45^ ^46^ The mechanism of action of hormonal treatment may be due to a regulatory effect on the endometrium.^44^ Tahara et al^46^ demonstrated a diminution and cessation of the spotting after three cycles of treatment with oral contraceptive pills in studied patients, and also observed a disappearance of scar dehiscences smaller than 3 mm after the treatment.

Florio et al^44^ compared the effectiveness of hysteroscopic correction and of hormonal treatment to improve symptoms associated with isthmocele. They showed that, compared with hormonal treatment, resectoscopic correction is more effective in shortening the duration of postmenstrual AUB and in reducing the prevalence of pelvic pain.^44^

Zhang et al^45^ evaluated operative and non-operative therapies, considering laparoscopy, vaginal repair, hysteroscopy, oral contraceptives, and levonorgestrel intrauterine system (LNG-IUS). All of the methods investigated, except for LNG-IUS, are useful in reducing the menstruation length in symptomatic patients.^45^

Therefore, oral contraceptive pills might represent a valid option for symptomatic women who do not want to get pregnant and prefer a conservative therapy.

#### Hysteroscopy

Hysteroscopy is the gold standard procedure for uterine cavity and cervical canal exploration and is the investigation of choice for AUB.^47^ ^48^ ^49^ During the hysteroscopy, the isthmocele appears as a bulging on the anterior wall of the cervical canal, easy to be localized on the isthmus site.^34^

Once diagnosed, an operative hysteroscopy can be performed to treat the defect, with a technique called isthmoplasty.

According to the literature, the essential parameter to perform hysteroscopy is the residual myometrial thickness; indeed, with the hysteroscopic approach, there is a risk of bladder injury and uterine perforation if the myometrium thickness at the site of the defect is < 3 mm.^50^ Some authors suggest hysteroscopy to women with a residual myometrial thickness > 2 to 2.5 mm or with a scar defect size to myometrial thickness ratio < 5 0% and with no desire to get pregnant.^51^ ^52^ ^53^

There is no homogeneous method to perform isthmoplasty, but almost every author uses a 9 mm resectoscope and unipolar electrical current. Gubbini et al^54^ performed a resection of the defect by removing the isthmocele edges and by putting its wall in continuity with the cervical canal wall. Fabres et al^55^ resected one edge of the scar and coagulated the thinnest part of the defect, allowing menstrual flow drainage to the cervix. Xie et al,^56^ who published one of the largest studies on isthmoplasty, performed it by simply removing the fibrotic tissue under the defect. As reported by Abacjew-Chmylko et al,^50^ some authors prefer to perform resectoscopy under ultrasonographic guidance, but this approach is not related to a lower morbidity rate.

According to the literature, the mean time for resectoscopic treatment varies from 8 to 25 minutes.^15^ ^51^ ^56^ Gubbini et al^57^ and Florio et al^58^ found an association between the duration of the isthmoplasty and the size of the niche.

The total amount of successful outcomes of isthmoplasty is 85.5% (59.6–100%).^15^ ^27^ ^51^ ^52^ ^53^ ^54^ ^55^ ^58^ An evident attenuation of the symptoms was associated not only with the removal of the scar diverticulum, in which the menstrual blood tends to be retained, but also with the fulguration of dilated vessels that constitute a potential additional source of non-menstrual bleeding.^50^

Good outcomes were also found regarding infertility: the majority of patients who desired to get pregnant conceived spontaneously between 12 and 24 months after the isthmoplasty.^54^ ^55^

According to Zhang et al,^45^ the comparison between hysteroscopy and medical treatment, intrauterine device (IUD), laparoscopy, and vaginal repair showed that hysteroscopic surgery offered the advantages of shorter operation time, reduced blood loss, decreased length of hospital stay, and lower hospital fees. However, one of the limitations of the resectoscopic treatment is the impossibility of the performance of sutures.^59^ This is why the scar defect could enlarge further, and the myometrial thickness at the level of the uterine isthmus could further decrease, increasing the risk of uterine rupture during future pregnancies.^60^

In the last few years, the development of new technologies applied to hysteroscopy have led to new interesting therapeutic applications of minimally invasive surgery for the treatment of many pathologies.^61^ ^62^ ^63^ Therefore, these new advances could change the way to approach the repair of isthmocele.

#### Vaginal Repair

Isthmocele vaginal repair has been evaluated by many authors.^45^ ^56^ ^59^ ^64^ ^65^ After identifying the defect as a small hollow area or depression at the uterine isthmus, thanks also to the guidance of a probe in the uterus, Chen et al^65^ performed a transverse incision at the most prominent area of the bulge; afterwards, the isthmocele was removed, and the edges of the incision were trimmed to repair it. Then, the myometrial and vaginal defects were closed. The median operation time was 33.6 minutes.^65^ Clinical improvement was observed in between 85.9 and 92.9% of the patients: the prolonged menstrual symptoms were improved after the surgery, and a significant difference was found between the mean preoperative and postoperative menstruation length.^59^ ^65^ Isthmocele transvaginal repair is comparably effective to the laparoscopic repair, but the surgical time is significantly shorter and the hospitalization expenses are lower.^45^

#### Laparotomy

With laparotomy, a complete resection of the dehiscent myometrium and an accurate uterine reconstruction can be performed.^25^ ^60^ ^66^ Pomorski et al^66^ proposed a minilaparotomy to patients who fulfilled three criteria: presence of symptoms, refusal of hormonal therapy, and residual myometrial thickness < 2.2 mm (this value increases the risk of uterine scar dehiscence or of rupture in a subsequent pregnancy). This cutoff value was chosen because if the thickness was larger, a less invasive method was to be preferred. The minilaparotomy of Pomorski et al^66^ was performed at the site of a previous CS. After the scar defect was identified, it was excised up to the endometrial layer of the anterior uterine wall, and, after that, the incision was closed. Shepker et al^60^ report a similar surgical technique but using a double-layered interrupted suture for uterine closure. Shepker et al^60^ recommend a complete abdominal resection of the niche and a reconstruction of the uterus by an exact adaptation of the margins of the wound in order to minimize the risk of subsequent uterine rupture.^60^ Laparotomy correction was successful in relieving postmenstrual spotting and abdominal pain, and a significant improvement in residual myometrial thickness was observed: preoperatively, the mean thickness was 1.9 mm, and 2 to 3 days after surgery, the mean thickness was 8.8 mm.^60^ ^66^ Jeremy et al^67^ described a pregnancy rate of 71% following the laparotomy procedure.

#### Laparoscopy

Since Jacobson et al^68^ first described the laparoscopic isthmocele resection in 2003, several authors adopted and described this surgical approach.^35^ ^45^ ^51^ ^64^ ^68^ ^69^ ^70^ ^71^ ^72^ ^73^ ^74^ ^75^ ^76^ ^77^ ^78^ ^79^ Laparoscopy is a technique that has to be preferred especially if the residual myometrial thickness is < 3 mm.^78^

A skilled laparoscopic surgeon can use conventional laparoscopy or robotic-assisted surgery to correct the isthmocele.^25^ After the defect is identified, it is cut open and the isthmocele and the surrounding fibrotic tissue are trimmed carefully and removed from the edges of the defect to access the healthy myometrium.^71^ Before closing, Donnez et al^35^ insert a Hegar probe into the cervix to preserve the continuity of the cervical canal with the uterus and perform a double-layer closure with separate sutures.

The critical step of the laparoscopic procedure is to correctly identify the isthmocele.^25^ This can be done using various techniques: easily laparoscopic visualization after dissecting the uterovesical peritoneum; hysteroscopy performed at the same time of the laparoscopy to evaluate the uterine cavity and the defect; moreover, the hysteroscopic transillumination better reveals the edges of the defect.^35^ ^51^ ^74^ Klemm et al^64^ recommended that if the scar was not immediately identifiable after the dissection of the uterovesical fold, a transvaginal sonography under laparoscopic view could be performed. Akdemir et al^76^ reported a case in which, during laparoscopy, a Foley catheter was used to identify the defect. The Foley catheter was inserted into the uterine cavity through the cervical canal, then it was filled at the lower uterine segment and, in this way, the isthmocele was clearly identified.^76^ Api et al^79^ described a technique named the “slip and hook technique”: since the defect could not be identified by laparoscopy and the light source of the laparoscope could not recognize the transillumination of the hysteroscopic light through the scar, a Hegar probe was placed in the cervical canal and then slipped forward blindly at the level of the uterine isthmus, bulging out the niche on the uterine wall. The continuing pressure on the defect led to a “hooking effect”, allowing its perforation under laparoscopic visualization.^79^ The surgical time varies between 42 and 90 minutes, and 240 minutes were needed for the robotic excision.^51^ ^70^ ^75^ ^79^

The laparoscopic approach treats symptoms by eliminating the reservoir effect of the defect, concomitantly strengthening the myometrial wall. In fact, Api et al^79^ analyzed the myometrial thickness, finding a mean value of 2 mm (0.7–6.2 mm) before the laparoscopy and of 9.8 mm (2.5–13.1 mm) after the surgery. Vervoort et al^78^ published the first large prospective cohort study that evaluated the effect of laparoscopic isthmocele resection on symptoms, on fertility, and on ultrasound findings, evaluating 101 women. In this study, Vervoort et al^78^ showed that the laparoscopic approach reduces postmenstrual spotting and its correlated discomfort, reduces dysmenorrhea, and enlarges the residual myometrial thickness 6 months after the intervention. The pregnancy rate after the laparoscopic approach is estimated to be 44%, as reported by Donnez et al.^35^

## Conclusion

A unified definition of CS defects should be formulated to have a unique, international terminology in order to avoid confusion in the literature. However, the main question of which surgical technique of CS diminishes the risk of scar development and its symptoms will probably remain unanswered. The clinical importance of isthmocele, however, relies on the diagnosis improvement with imaging tools. Every time we suspect the presence of isthmocele in a patient with at least one CS in her history, the first diagnostic approach should be performed with TVUS and SHG, especially in those patients with AUB, pelvic pain, infertility, and dysmenorrhea. Isthmocele treatment should be based on the history of the symptoms of the patient, on, the desire of future pregnancy, and on the characteristics of the isthmocele. Therefore, it is very important to discuss the management with the patient, although it is not possible to speculate which treatment appears to be superior to the other. It is important to highlight that treatment should be proposed only to symptomatic patients. Isthmocele is a very fertile and actual field of research due to its increased rate. Further studies are needed to prevent its development and to increase the efficacy of its management.
